# Mouse polyomavirus infection induces lamin reorganisation

**DOI:** 10.1111/febs.17275

**Published:** 2024-09-17

**Authors:** Kateřina Bruštíková, Boris Ryabchenko, Sandra Žáčková, Vojtěch Šroller, Jitka Forstová, Lenka Horníková

**Affiliations:** ^1^ Department of Genetics and Microbiology, BIOCEV, Faculty of Science Charles University Prague Czech Republic; ^2^ Present address: Virology Department, Institute of Organic Chemistry and Biochemistry Czech Academy of Sciences Prague Czech Republic

**Keywords:** lamin A/C, lamin B, mouse polyomavirus, viral replication centres, VP1

## Abstract

The nuclear lamina is a dense network of intermediate filaments beneath the inner nuclear membrane. Composed of A‐type lamins (lamin A/C) and B‐type lamins (lamins B1 and B2), the nuclear lamina provides a scaffold for the nuclear envelope and chromatin, thereby maintaining the structural integrity of the nucleus. A‐type lamins are also found inside the nucleus where they interact with chromatin and participate in gene regulation. Viruses replicating in the cell nucleus have to overcome the nuclear envelope during the initial phase of infection and during the nuclear egress of viral progeny. Here, we focused on the role of lamins in the replication cycle of a dsDNA virus, mouse polyomavirus. We detected accumulation of the major capsid protein VP1 at the nuclear periphery, defects in nuclear lamina staining and different lamin A/C phosphorylation patterns in the late phase of mouse polyomavirus infection, but the nuclear envelope remained intact. An absence of lamin A/C did not affect the formation of replication complexes but did slow virus propagation. Based on our findings, we propose that the nuclear lamina is a scaffold for replication complex formation and that lamin A/C has a crucial role in the early phases of infection with mouse polyomavirus.

## Graphical abstract

Here, we investigated the roles of lamins as important nuclear proteins in the replication cycle of the mouse polyomavirus. We detected accumulation of the major capsid protein VP1 under the nuclear lamina, defects in nuclear lamina staining and different lamin A/C phosphorylation patterns following viral infection. Additionally, lamins were found in virus replication centres, and the absence of lamin A/C slowed virus replication, suggesting that lamin A/C may play a regulatory role in this process.

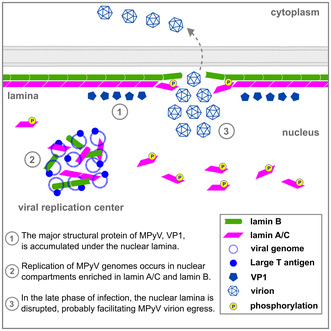

**Abbreviations**
BKPyV: BK polyomavirus
FISH: fluorescence in situ hybridisation
hpi: hours post‐infection
JCPyV: JC polyomavirus
LADs: lamin‐associated domains
LT: large tumour antigen
MOI: multiplicity of infection
MPyV: mouse polyomavirus
PARP‐1: poly(ADP‐ribose) polymerase‐1
PCNA: proliferating cell nuclear antigen
pfu: plaque forming unit
STED: stimulated emission depletion
SV40: Simian virus 40
VP1: major capsid protein
VP2/3: minor capsid protein
VRCs: viral replication centres

## Introduction

Nuclear lamina, which is the main component of the nuclear cytoskeleton, is a protein meshwork adjacent to the nucleoplasmic side of the inner nuclear membrane and is composed of the type V intermediate filament proteins called nuclear lamins [1, 2]. There are two types of lamins: A‐type and B‐type. A‐type lamins (A and C lamin isoforms) are generated by alternative splicing of the primary transcript of a single *LMNA* gene and are produced almost exclusively in differentiated cells. B‐type lamins (the most common B1 and B2 lamins) are encoded by distinct *LMNAB1* and *LMNAB2* genes ubiquitously expressed in embryonic and differentiated cells. Both types of lamins form independent but closely cooperating protein networks [3, 4]. These networks serve as a scaffold for the nuclear envelope and chromatin anchoring sites, thereby defining and maintaining the shape and mechanical stability of the nucleus and spatial organisation of chromatin in the interphase nucleus. In addition, lamins interact with numerous proteins, including nuclear membrane proteins, signalling molecules, chromatin and its regulators and various transcription and splicing factors. Therefore, lamins have a crucial role in different cellular processes such as cell division, DNA replication and repair, RNA transcription and epigenetic modification or spatial organisation of chromatin and mechano‐signalling [5, 6].

The nuclear lamina may also be a physical barrier to virus replication. Mouse polyomavirus (MPyV) is a small dsDNA virus from the family *Polyomaviridae*, which is a group of non‐enveloped tumorigenic viruses that replicate in the cell nucleus. The MPyV genome is a circular dsDNA molecule that, together with cellular histones (except H1), forms a nucleocore surrounded by an icosahedral capsid composed of three structural proteins: the major capsid protein VP1, and the minor capsid proteins VP2 and VP3. During its replication cycle, MPyV crosses the nuclear envelope twice – first, when entering the nucleus at the beginning of the infection and second, when the viral progeny escapes the nucleus of infected cells to initiate a new infection cycle. MPyV virions enter host cells via endocytosis initiated through the interaction of VP1 protein with ganglioside receptor GD1a or GT1b [7]. Subsequently, for a productive infection, virions are transported along microtubules using early and late endosomes to the endoplasmic reticulum [8, 9]. In the endoplasmic reticulum, partial disassembly of virions exposes hydrophobic sequences of VP2 and VP3 and leads to the translocation of incompletely disassembled virions to the cytosol [10, 11]. There, importins recognise a nuclear localisation signal of the structural proteins and the virions are transported through nucleopores to the cell nucleus [12, 13]. In the nucleus, the early region of the virus genome, which encodes antigens called T (tumour) antigens, is transcribed first. Large, middle and small T antigens – essential for virus propagation – are translated from mRNAs generated from an alternatively spliced common transcript. The T antigens interact with multiple cellular pathways that culminate in the host cells entering the S phase of the cell cycle to ensure a suitable environment for the progression of the infection [14, 15]. In addition, the large T (LT) antigen is involved in the transcription of viral and cellular genes and is indispensable for the initiation of genome replication [14]. Replication of viral genomes induces intensive transcription of the late structural proteins VP1, VP2 and VP3 and assembly of new virions. Viral progeny are partially released from the nucleus and cells actively by an as yet unclear process and then passively after cell death.

Although several viruses have evolved diverse mechanisms to circumvent the nuclear lamina (reviewed in ref. [16]), knowledge of polyomaviruses is limited. In cells infected with human JC polyomavirus (JCPyV), the nuclear envelope was found to be destabilised in the late phase of infection. The non‐structural protein expressed in the late phase of JCPyV infection – agnoprotein – binds heterochromatin protein 1, leading to increased mobility of lamin B receptor and subsequent destabilisation of the nuclear envelope. These changes facilitate the nuclear egress of viral particles in infected cells [17]. Conversely, in cells infected with simian virus 40 (SV40), which is a polyomavirus that infects primates, changes in the morphology of the nuclear envelope and fluctuations in lamin A/C protein levels were observed at early times post‐infection. These changes were connected with partial cleavage of lamin A/C by caspase‐6 but did not lead to increased permeability of the nuclear envelope. Since the peak of these changes appeared during the entry of the viral genome into the nucleus, alternative trafficking of the SV40 genome from the endoplasmic reticulum to the nucleus through virus‐induced nuclear lamina remodulations was postulated [18].

In this study, we focused on the role of nuclear lamina in the late phase of MPyV infection. In addition, we studied the impact of lamin A/C deficiency on virus propagation. We detected VP1 accumulation under the nuclear lamina, staining defects of the lamina, and different lamin A/C phosphorylation states, but the nuclear envelope remained intact in the late phase of infection. Furthermore, a lack of lamin A/C did not affect replication complex formation but did decelerate virus propagation. Thus, lamin A/C appears to have played a key role in the early phases of infection. Based on our findings, we propose that lamins are the scaffold for the formation of polyomavirus transcription/replication complexes.

## Results

### Newly synthetised VP1 accumulates at the nuclear periphery under nuclear lamina in the late phase of MPyV infection

**Fig. 1:**
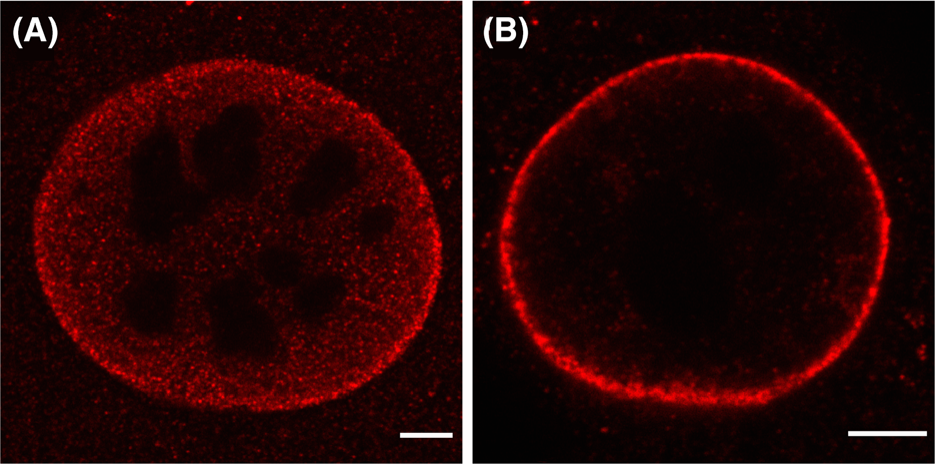
Two VP1 staining patterns are detected in the nucleus of infected 3T6 cells; a diffuse nucleoplasmic localisation (A) and accumulation at the nuclear periphery (B). Mouse 3T6 cells were infected with MPyV (MOI = 1 pfu per cell), fixed at 32 h post‐infection and VP1 was stained using a specific antibody. Images represent selected confocal sections of the indicated cells. Bar, 5 μm.

Localisation of VP1 protein in the nucleus of infected 3T6 cells was analysed by confocal microscopy. Cells were infected with MPyV, fixed at 32 h post‐infection (hpi) and stained with a specific antibody against VP1. Two VP1 staining patterns were observed in the cell nucleus: a diffuse localisation of VP1 throughout the nucleoplasm with obvious accumulation at the nuclear periphery (Fig. 1A) or localisation at the nuclear periphery in a ring formation, suggesting accumulation of VP1 under the nuclear lamina (Fig. 1B).

**Fig. 2:**
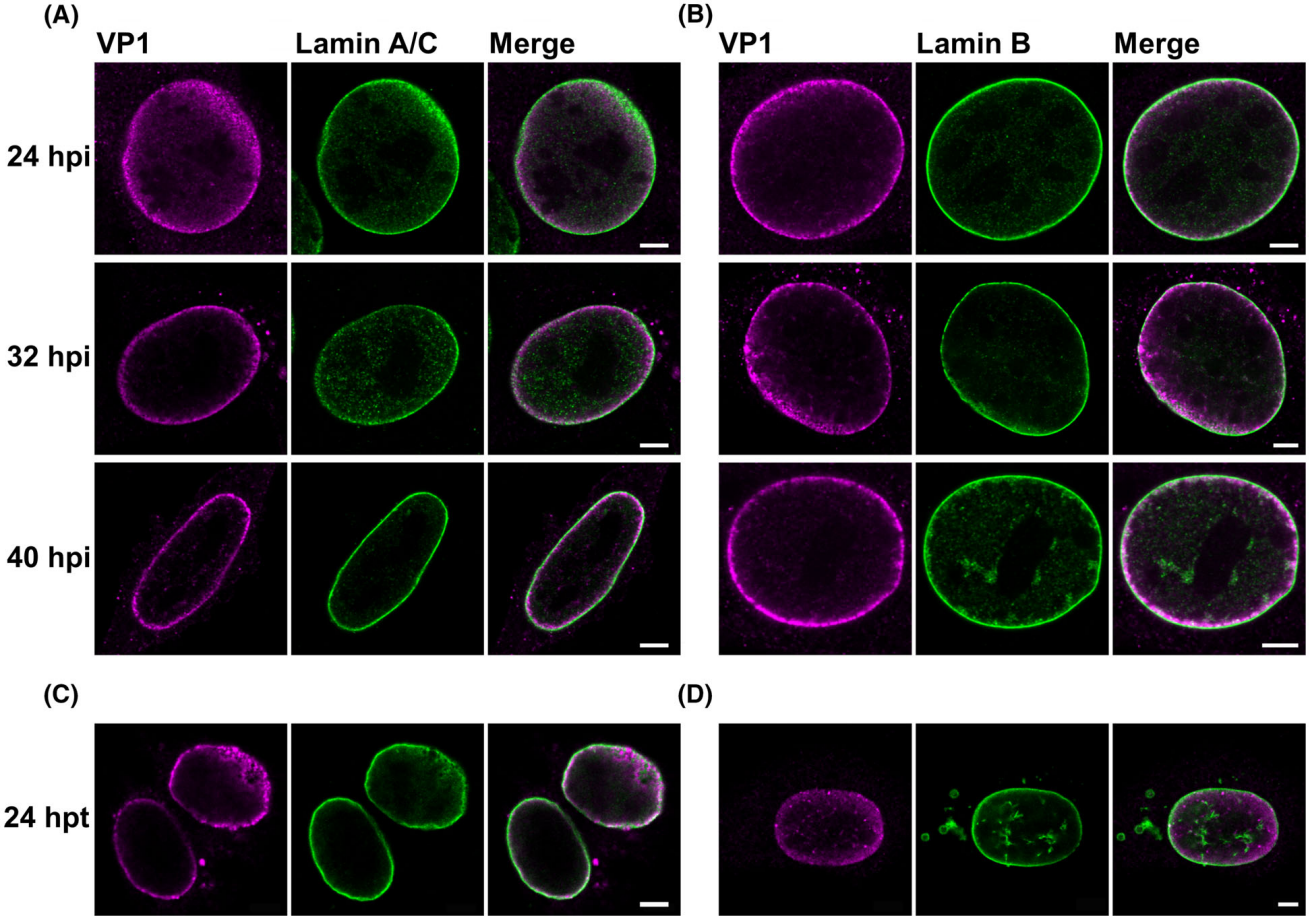
VP1 accumulates at the nuclear periphery during the late phase of infection. (A, B) Mouse 3T6 cells were infected with MPyV, and at the indicated hours post‐infection (hpi), VP1 (magenta) and lamin A/C (green; A) or lamin B1 (green; B) were stained with specific antibodies. (C, D) Mouse NIH‐3T3 fibroblasts were transfected with plasmid pEF1‐LATE, and 24 h post‐transfection (hpt), VP1 (magenta) and lamin A/C (green; C) or lamin B1 (green; D) were stained with specific antibodies. Images represent selected confocal sections of the indicated cells. Bar, 5 μm.

**Fig. 3:**
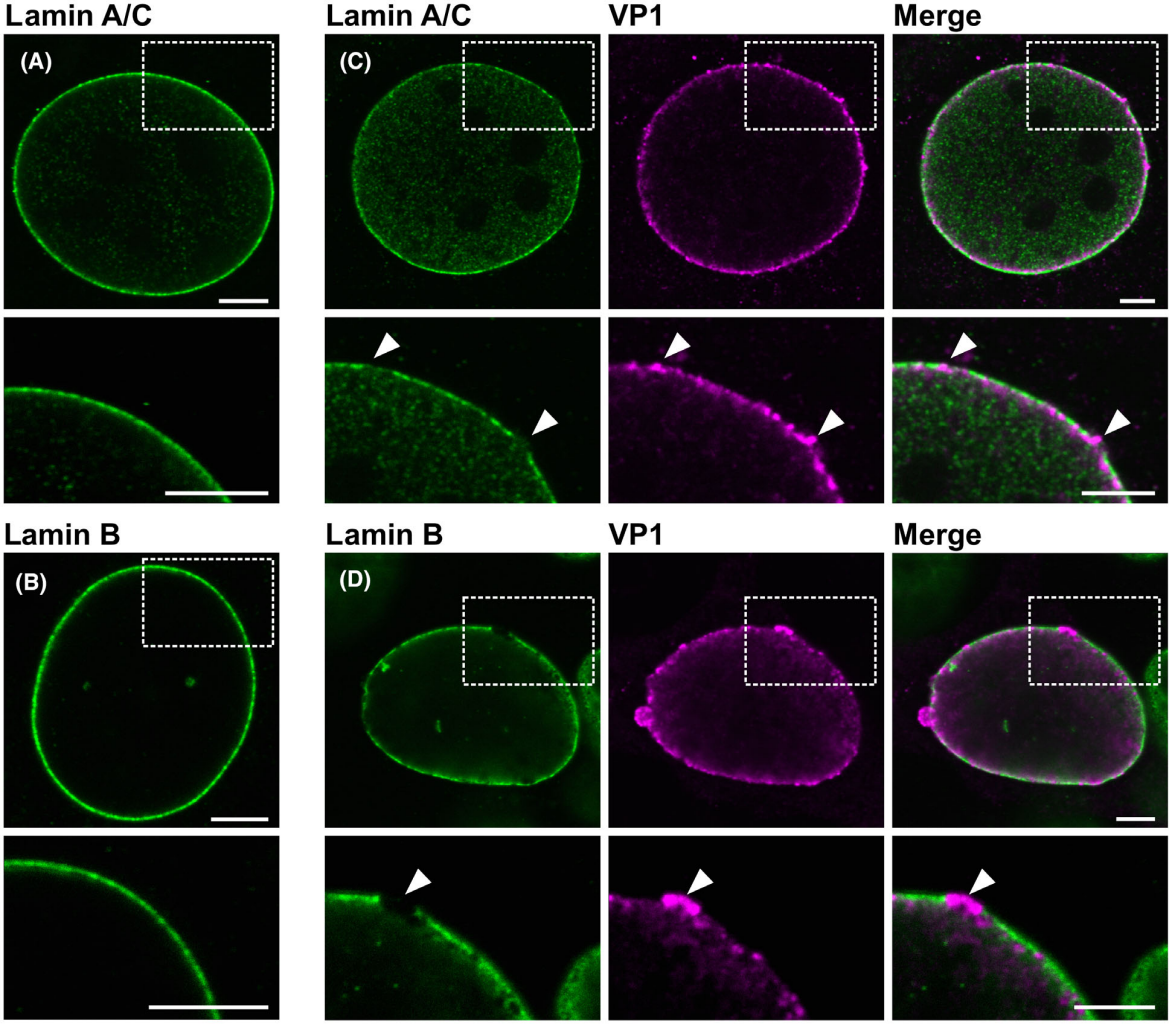
Depletions in lamin staining are detected in the late phase of infection. Mouse 3T6 cells were mock‐infected (A, B) or infected with MPyV (C, D), and at 40 h post‐infection, VP1 (magenta) and lamin A/C (green; A, C) or lamin B1 (green; B, D) were stained using specific antibodies. Images represent selected confocal sections of indicated cells. Enlarged details of the cells, highlighted by the dashed boxed regions in the upper panels, are presented in the lower panels. Bar, 5 μm.

To examine whether the distribution of VP1 in the nucleus changes during the late phase of MPyV infection and whether VP1 accumulates in close proximity of the nuclear lamina, cells were infected with MPyV, fixed at 24, 32 or 40 hpi and stained with antibodies against VP1 and lamin A/C or lamin B1 for confocal microscopy. Both VP1 staining patterns were observed at 24 hpi, but at later times post‐infection, the VP1 signal was predominantly detected at the nuclear periphery and this phenotype completely prevailed at 40 hpi (Fig. 2). VP1 localised in close proximity to the nuclear lamina but no abundant colocalisation of VP1 with either lamin A/C or lamin B1 was detected (Fig. 2). However, in approximately 20% of the infected cells fixed at 40 hpi, lamin A/C staining was depleted (compared with mock‐infected cells) and a strong VP1 signal was present at the sites of depletion (Fig. 3A,C). Depleted lamin B1 staining was also detected in infected cells, but this phenotype was rare (< 1%). At the sites of depletion, a strong VP1 signal protruding from the nucleus was present (Fig. 3B,D).

A similar staining pattern of VP1 was detected in cells transiently expressing all capsid proteins VP1, VP2 and VP3 (Fig. 2C,D). Similar to the infected cells, VP1 was detected at the nuclear periphery, lamin A/C or lamin B1 staining was depleted in some cells and in the sites of depletion only VP1 signal was observed (Fig. 2C,D).

These observations indicated a possible loss of integrity of the nuclear lamina followed by the breakdown of the nuclear envelope, as demonstrated for several virus families (reviewed in ref. [16]).

### Levels of lamin A/C and lamin B1 are altered during MPyV infection

**Fig. 4:**
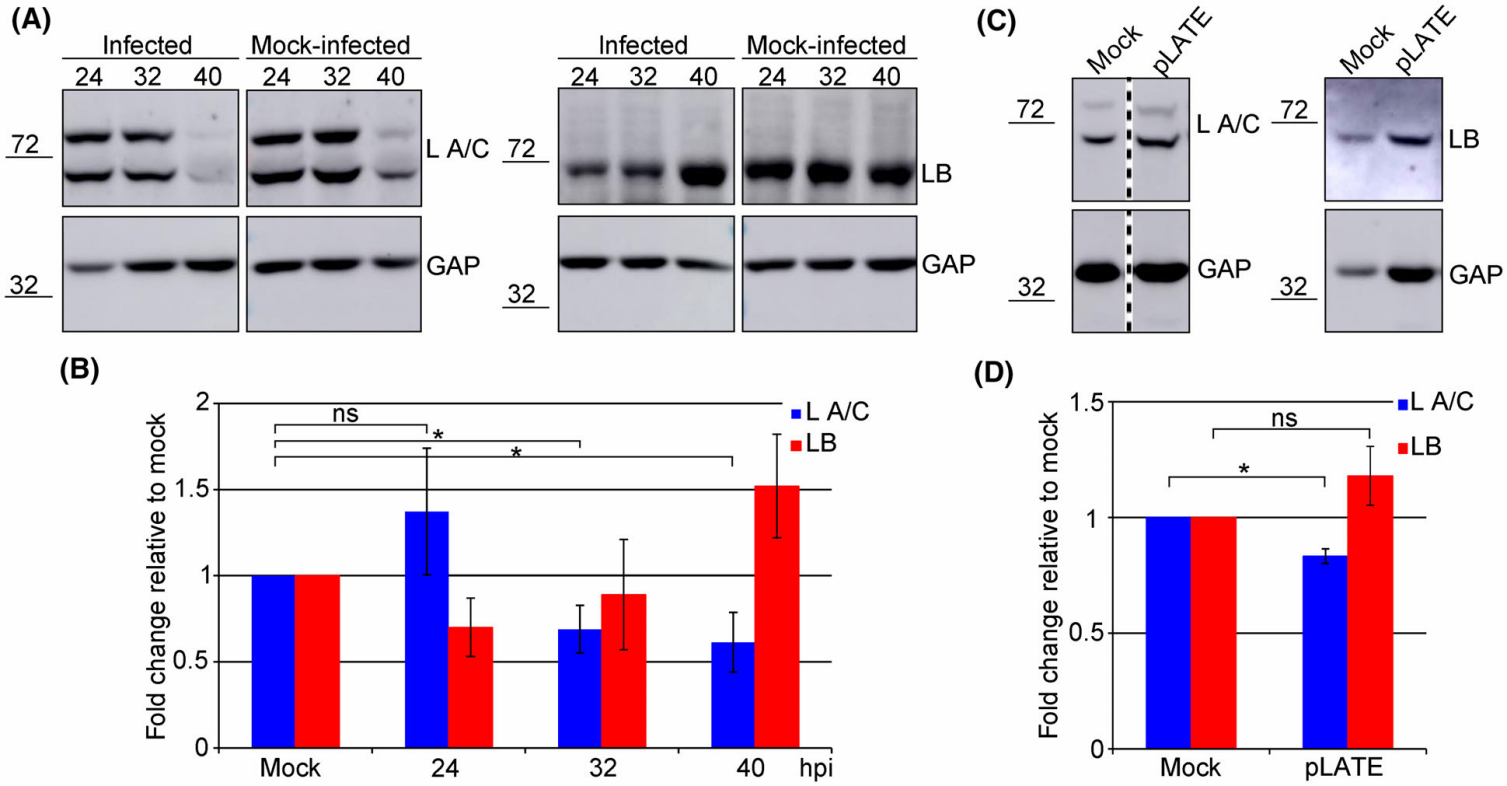
Lamin A/C and lamin B1 levels are altered in infected cells. (A) Mouse 3T6 cells were infected and lysed at the indicated hours post‐infection (hpi). (C) WOP cells were transfected with plasmid pEF1‐LATE (pLATE) and lysed 24 h post‐transfection. Lysates were separated by SDS/PAGE, transferred onto a nitrocellulose membrane, and lamin A/C (L A/C), lamin B1 (LB) and GAPDH (GAP) were detected using specific antibodies. Some of the blots were spliced as indicated by the vertical dashed lines. (B, D) Graphic illustration of densitometry analysis of the digital images of western blots from three (D) or four (B) independent experiments. The data show the fold increase relative to mock‐infected or mock‐transfected cells. Error bars represent standard deviation. *P < 0.05 determined by Student's *t*‐test. Changes in total levels of lamin B1 (B, red columns) were not significant (ns) according to Student's *t*‐test.

To determine the source of the observed staining depletions, we tracked the levels of lamin A/C and lamin B1 in infected cells. Mouse fibroblasts were infected with MPyV, lysed at 24, 32 or 40 hpi and levels of lamin A/C and lamin B1 were analysed by western blot. Although the level of lamin A/C in cells lysed at 24 hpi was slightly elevated compared with that in mock‐infected cells, at later times post‐infection, the amount of lamin A/C decreased gradually up to 61% at 40 hpi compared with that in mock‐infected cells (Fig. 4A,B). In contrast, lamin B1 showed the opposite trend. At 24 hpi, the level of lamin B1 was reduced to 70% of that in control cells, and at later times post‐infection, the level of lamin B1 increased gradually up to 152% at 40 hpi compared with that in mock‐infected cells (Fig. 4A,B). These observed alterations were not due to regulation of host gene expression during virus infection as we did not detect any significant changes in lamin A/C and lamin B1 mRNA levels (data not shown). Analogous results were obtained in cells transiently expressing structural proteins. In these cells, the level of lamin A/C was reduced to 83% in comparison with control cells and the amount of lamin B1 was increased to 118% in comparison with control cells (Fig. 4C,D). These data support the hypothesis that the nuclear lamina is affected at late times post MPyV infection.

### The nuclear envelope is not disrupted at late times post‐infection

Several methods were used to explore whether the nuclear lamina and nuclear envelope were disrupted at late times post‐infection with MPyV. As disrupted staining of the nuclear lamina was observed at 40 hpi and the most significant change in lamin amounts was detected at the same time point, the following experiments were conducted at 40 hpi.

**Fig. 5:**
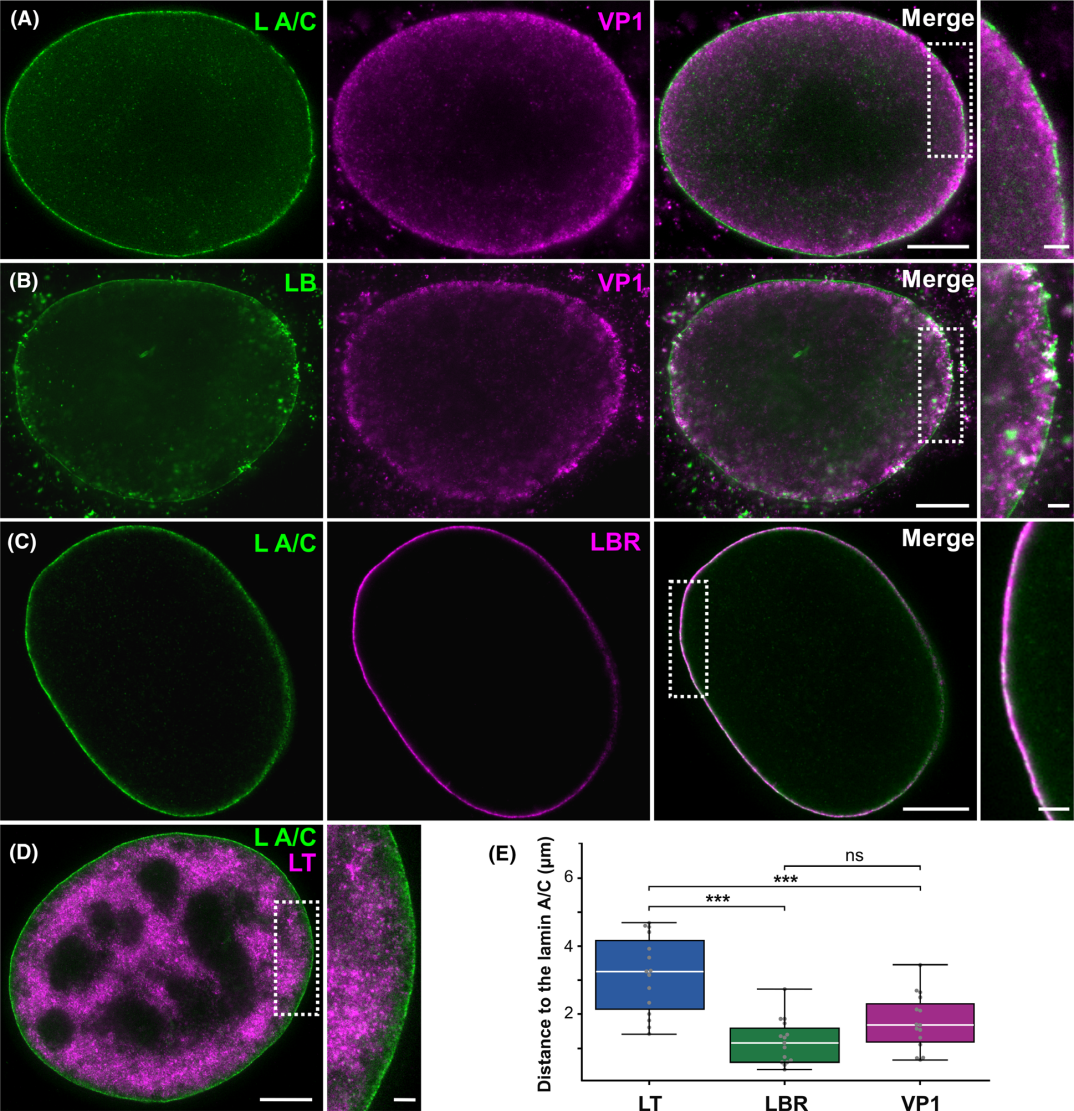
VP1 accumulates nonrandomly in close proximity to the nuclear lamina. Mouse 3T6 cells were infected with MPyV (MOI = 1 pfu per cell) and fixed at 40 h post‐infection. (A) VP1 (magenta) and lamin A/C (L A/C; green), (B) VP1 (magenta) and lamin B1 (LB; green), (C) lamin A/C (green) and lamin B receptor (LBR; magenta) or (D) lamin A/C (green) and LT antigen (magenta) were stained using specific antibodies. Bar, 5 μm. Enlarged details of the cells, indicated by dashed boxed regions, are presented in side panels. Bar, 1 μm. (E) The distance of VP1, LBR (positive control) and LT (negative control) from the border of the nucleus defined by lamin A/C was measured. Each point represents the mean value of distances from one nucleus; the original data resembled log‐normal distribution. White lines represent median values; the bottom and top edges of the box indicate the 25th and 75th percentiles, respectively. The whiskers extend from the box by 1.5× the interquartile range. Outliers would be shown if present. *P*‐values were obtained by Tukey HSD test following analysis of variance (ANOVA) (*P*‐value of ANOVA is < 0.000001). ***P < 0.001; ns, not significant. Sample size, *n* = 15. Samples from groups with more data were randomly sampled using equal probabilities and with no replacement.

First, a more detailed analysis of the nuclear lamina in the infected cells was performed using stimulated emission depletion (STED) microscopy. This technique uses a STED super‐resolution nanoscope with high detection efficiency of far‐red photons to distinguish VP1 signals of individual particles in the STED channel and thus visualise VP1 and the nuclear lamina in high resolution. Mouse fibroblast 3T6 cells were infected, then VP1 and lamin A/C or lamin B1 were stained with appropriate antibodies and visualised by the 2D super‐resolution STED technique. As expected, disrupted staining of lamin A/C and lamin B1 was observed in the infected cells (Fig. 5A,B). Comparable to the confocal microscopy analysis, disrupted lamin A/C staining was detected in 25% of cells (*n* = 84) and depleted lamin B1 staining was detected in 5% of cells (*n* = 35). VP1 protein was found in close proximity to lamin B1 and was tightly associated with lamin A/C (Fig. 5A,B). Although signal co‐localisation analysis of the confocal microscopy images revealed high values of Pearson's coefficient, indicating potential co‐localisation of VP1 with both lamins, analysis of the STED images did not confirm this co‐localisation. These data indicate that VP1 and lamin A/C or lamin B1 are localised close together but do not overlap.

To exclude random, sterically conditioned accumulation of VP1 at the nuclear periphery, the distance of VP1 protein from the inner part of the nuclear lamina – lamin A/C – was determined. Subsequently, the mean distance of the VP1–lamin A/C signal was compared with that of LT–lamin A/C (negative control). LT is distributed throughout the nucleus and does not obviously accumulate under the nuclear lamina. If there was sterically conditioned accumulation of VP1 in close proximity to the nuclear lamina, the mean distance of VP1 and lamin A/C would be comparable with the mean distance of LT and lamin A/C. Lamin B receptor, which is a protein embedded in the inner nuclear membrane and thus located in close proximity to the nuclear lamina, was used as a positive control. The signals of lamin B receptor and lamin A/C had a high Pearson's correlation coefficient (approximately 0.61) and the mean distance of these signals was 1.18 μm. However, LT antigen and lamin A/C had a low Pearson's correlation coefficient value (approximately 0.19), and the LT signal was found further from the lamin A/C signal, with a mean distance of 3.13 μm. Although VP1 and lamin A/C had a low Pearson's correlation coefficient value (approximately 0.22), comparable with that for LT antigen, these two signals were detected in close proximity to each other, with a mean distance of 1.84 μm (Fig. 5). These data indicate that the accumulation of VP1 in close proximity to the nuclear lamina is not random.

**Fig. 6:**
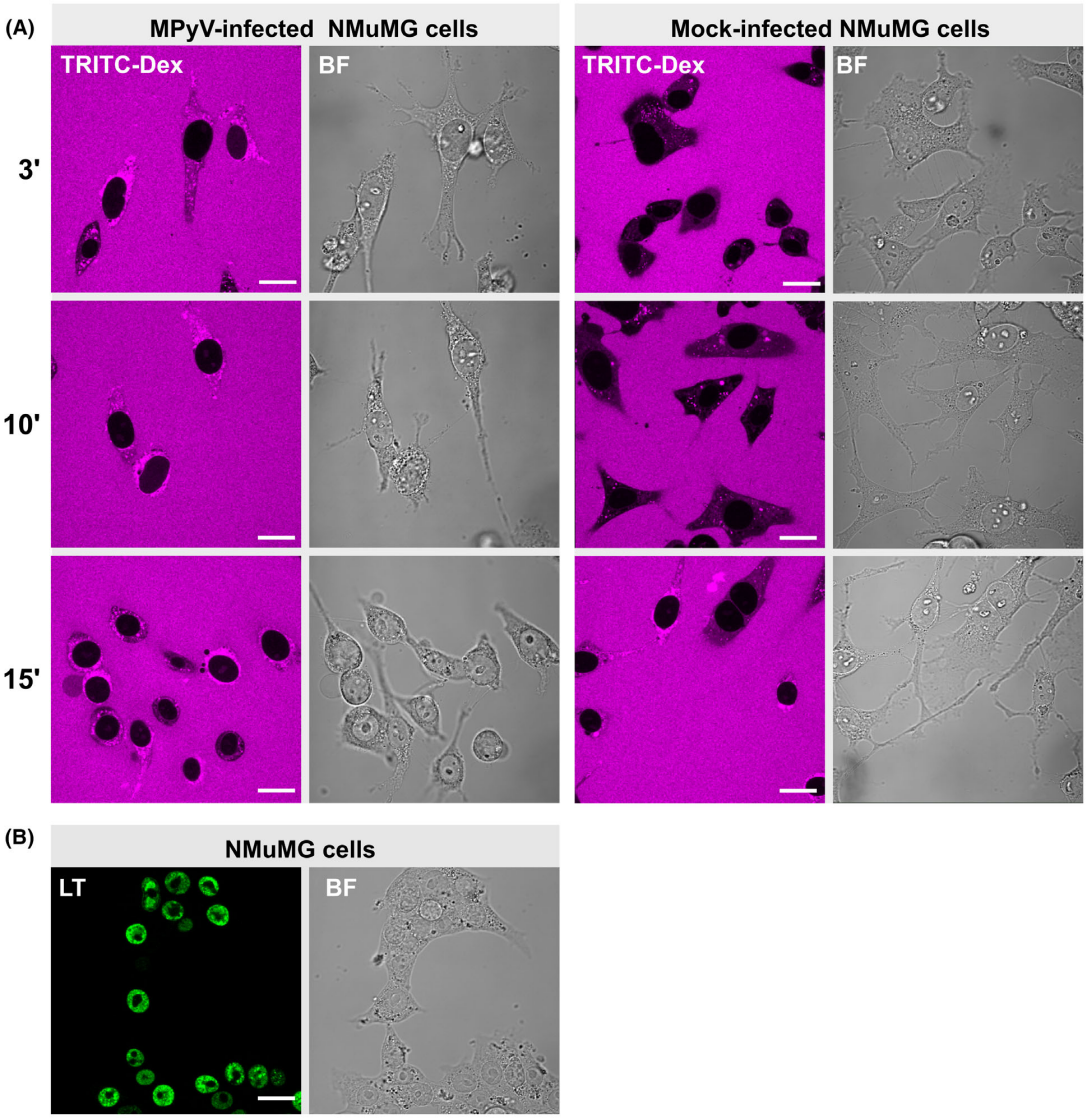
The nuclear envelope remains intact in MPyV‐infected cells. (A) NMuMG cells were mock‐infected or infected with MPyV (MOI = 10 pfu per cell), and at 40 h post‐infection, the cells were permeabilised with digitonin and incubated with high‐molecular‐weight dextran (155 kDa) conjugated with TRITC (TRITC‐Dex) (magenta). Confocal sections of live cells at indicated times after TRITC‐Dex addition are presented (in minutes). (B) In parallel, to confirm infection of the cells, the infected NMuMG were fixed and then LT antigen (green) was stained using specific antibodies. BF, bright field. Bar, 20 μm.

To further evaluate the integrity of the nuclear lamina and envelope, the semi‐permeabilised cell assay was applied. If the nuclear envelope permeability was affected in infected cells, we should detect an influx of high‐molecular‐weight dextran into the cell nucleus. Mouse epithelial NMuMG cells were used in this assay. These cells, which are permissive for MPyV, are round and have a smaller cytoplasmic volume than 3T6 fibroblasts, allowing rapid diffusion of the high‐molecular‐weight dextran to the proximity of the cell nucleus. NMuMG cells were infected with MPyV, the plasma membrane was permeabilised by digitonin at 40 hpi and high‐molecular‐weight (155‐kDa) tetramethyl rhodamine isocyanate (TRITC)‐labelled dextran was added to the cells. The nuclear influx of the TRITC‐labelled dextran was monitored using live cell microscopy for 15 min. In parallel, some cells were fixed and stained with a specific antibody against LT antigen to confirm that all cells were infected. In control and infected cells, TRIC‐labelled dextran accumulated in the cytoplasm close to the nucleus. With a few exceptions that could be caused by natural disassembly of the nuclear envelope at the onset of mitosis or apoptosis, we did not detect an influx of TRITC‐labelled dextran into the nuclei of infected or control cells even after 15 min (Fig. 6).

Collectively, these data demonstrate that VP1 accumulates in close proximity to the nuclear lamina in a non‐random manner. The nuclear lamina is disturbed in infected cells at late times post‐infection, but the nuclear envelope remains intact.

### Lamin A/C solubility is increased in MPyV‐infected cells

**Fig. 7:**
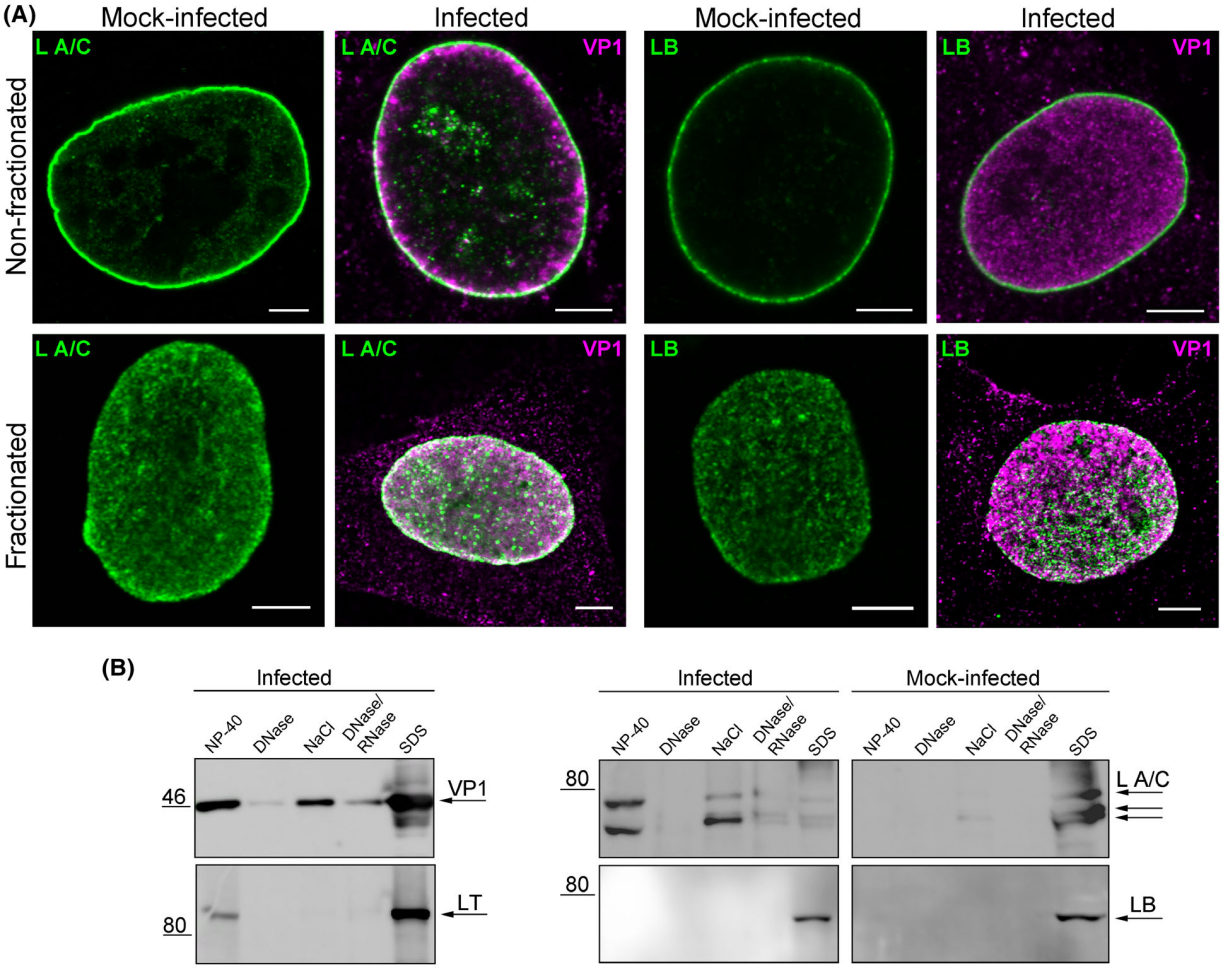
Lamin A/C is solubilised in infected cells. (A) Mouse 3T6 cells were infected, fractionated *in situ* at 40 h post‐infection (hpi) and VP1 (magenta) and lamin A/C (L A/C; green) or lamin B1 (LB; green) were stained with specific antibodies. Images are selected confocal sections of non‐fractionated cells or cells after the final fractionation step. Bar, 5 μm. (B) Mouse 3T6 cells were infected, fractionated *in situ* at 40 hpi and washed‐out material from each fraction was separated by SDS/PAGE and transferred onto a nitrocellulose membrane. The presence of VP1, LT, lamin A/C and lamin B1 in each washed‐out fraction was determined using specific antibodies.

Although infection of cells with MPyV did not extensively disrupt the lamina, minor reorganisations of the lamina were detected in these cells. These reorganisations may manifest as changes in the solubility of lamins. Therefore, we employed *in situ* fractionation to examine potential changes in the solubility of lamins in infected cells. *In situ* fractionation is designed to study nuclear matrix proteins without altering the morphology of non‐extracted cellular structures [19, 20]. The method is based on successive washing out of proteins according to their solubility in different buffers and allows analysis of the proteins washed out in every step as well as analysis of the cellular structures remaining attached to the culture dish. First, the residual structures after *in situ* fractionation were analysed by fluorescence microscopy. Despite the fact that lamins are predominantly found at the nuclear lamina, a portion of A‐type lamins is found inside the nuclear interior where it interacts with chromatin and participates in gene regulation [6]. A fraction of B‐type lamins is also suggested to be present in the nuclear interior [21]. We anticipated that stepwise washing out of proteins might reveal hidden epitopes, and potential complexes of lamins and VP1 would be detectable as a result. Mouse fibroblasts were infected with MPyV and subjected to *in situ* fractionation at 40 hpi. Subsequently, VP1, lamin A/C and lamin B1 were stained in non‐fractionated and fractionated cells using specific antibodies. *In situ* fractionation revealed a significant increase of lamin A/C inside the nucleus of infected and control cells and a strong degree of colocalisation of VP1 and lamin A/C signals. Lamin B1 was also detected inside the nucleus of infected and control cells, but only negligible colocalisation of VP1 and lamin B1 was observed in the infected cells. Instead, VP1 and lamin B1 signals were juxtaposed at the nuclear periphery and inside the nucleus (Fig. 7A). These data indicate that nucleoplasmic lamin A/C may be a part of VP1 complexes.

Next, the wash‐out fractions were analysed. As highly insoluble proteins, lamins should be exclusively found in the last highly insoluble fraction [19]. We expected that reorganisation of the lamins would change the solubility of individual lamin species, resulting in their detection in soluble fractions. Mouse fibroblasts were infected with MPyV, subjected to *in situ* fractionation at 40 hpi and the presence of VP1, LT antigen, lamin A/C and lamin B1 was tested in each fraction (Fig. 7B). VP1 protein was found in all fractions, with a marked abundance in the last highly insoluble fraction (SDS fraction). LT antigen was detected in the soluble fraction (NP‐40 fraction) and in the last highly insoluble fraction (SDS fraction). Lamin B1 was found exclusively in the SDS fraction of infected and control cells suggesting that no solubilisation of lamin B1 had occurred. In mock‐infected cells, lamin A/C was detected predominantly in the insoluble SDS fraction and a trace amount was found in the soluble NaCl fraction (Fig. 7B). However, in infected cells, lamin A/C was detected in almost all fractions (except the DNase fraction) and predominantly in the soluble NP‐40 fraction (Fig. 7B).

**Fig. 8:**
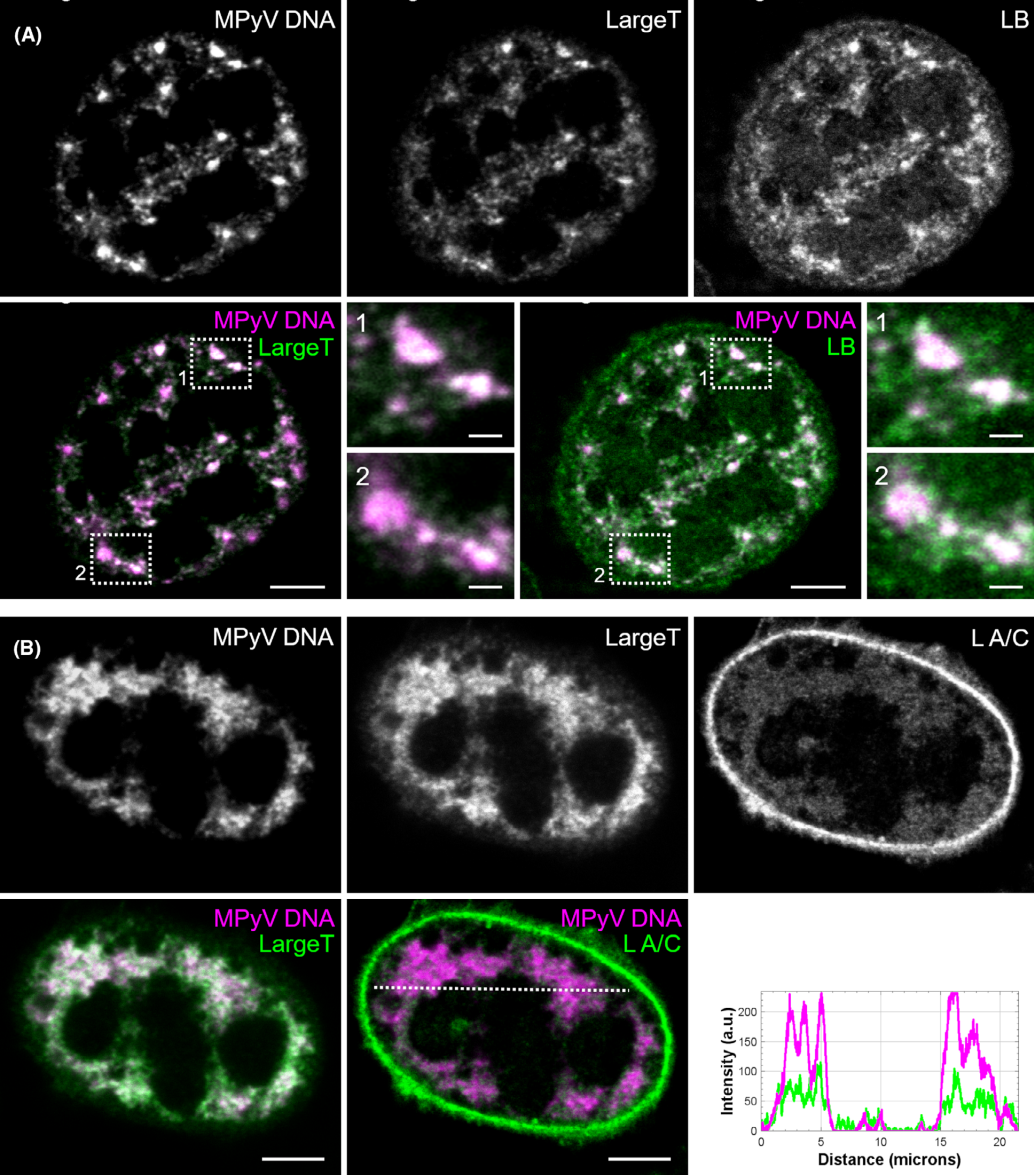
Viral replication centres are associated with nuclear lamins. Mouse 3T6 cells were infected, and at 40 h post‐infection, viral DNA was labelled by fluorescence *in situ* hybridisation (magenta) and large T antigen (green) and lamin B1 (LB; green) (A) or lamin A/C (L A/C; green) (B) were stained with specific antibodies. Bar, 5 μm. Enlarged details of the cells, indicated by dashed boxed regions, are presented in side panels. Bar, 1 μm. Images are selected confocal sections of the indicated cells. The graph represents the intensities of MPyV DNA and lamin A/C signals corresponding to the dotted line in the last image.

Since we detected LT antigen in the insoluble SDS fraction, together with lamin A/C and lamin B1, we assumed that these proteins may be associated with complexes in viral replication centres (VRCs). Therefore, we wondered whether viral DNA was a component of these presumed complexes. To characterise such complexes, mouse fibroblasts were infected with MPyV for 40 h, then viral DNA was labelled by fluorescence *in situ* hybridisation (FISH) and LT, lamin A/C or lamin B1 were stained using specific antibodies. Colocalisation of MPyV DNA with LT was observed and distinct spots likely representing VRCs were detected (Fig. 8). Although the FISH technique slightly affected the staining of the lamins, the lamin B1 signal colocalised with LT and MPyV DNA complexes, and a weak lamin A/C signal colocalising with LT and MPyV DNA signals was also revealed (Fig. 8).

These data show that solubilisation of lamin A/C is induced in the late phase of infection, supporting the hypothesis that the nuclear lamina is locally disrupted. Furthermore, our findings suggest that VP1 and LT can be associated with nuclear lamins. MPyV genomes are part of the complexes containing LT tightly associated with nuclear lamins and these complexes apparently represent VRCs.

### A different phosphorylation pattern of lamin A/C is detected in infected cells

**Fig. 9:**
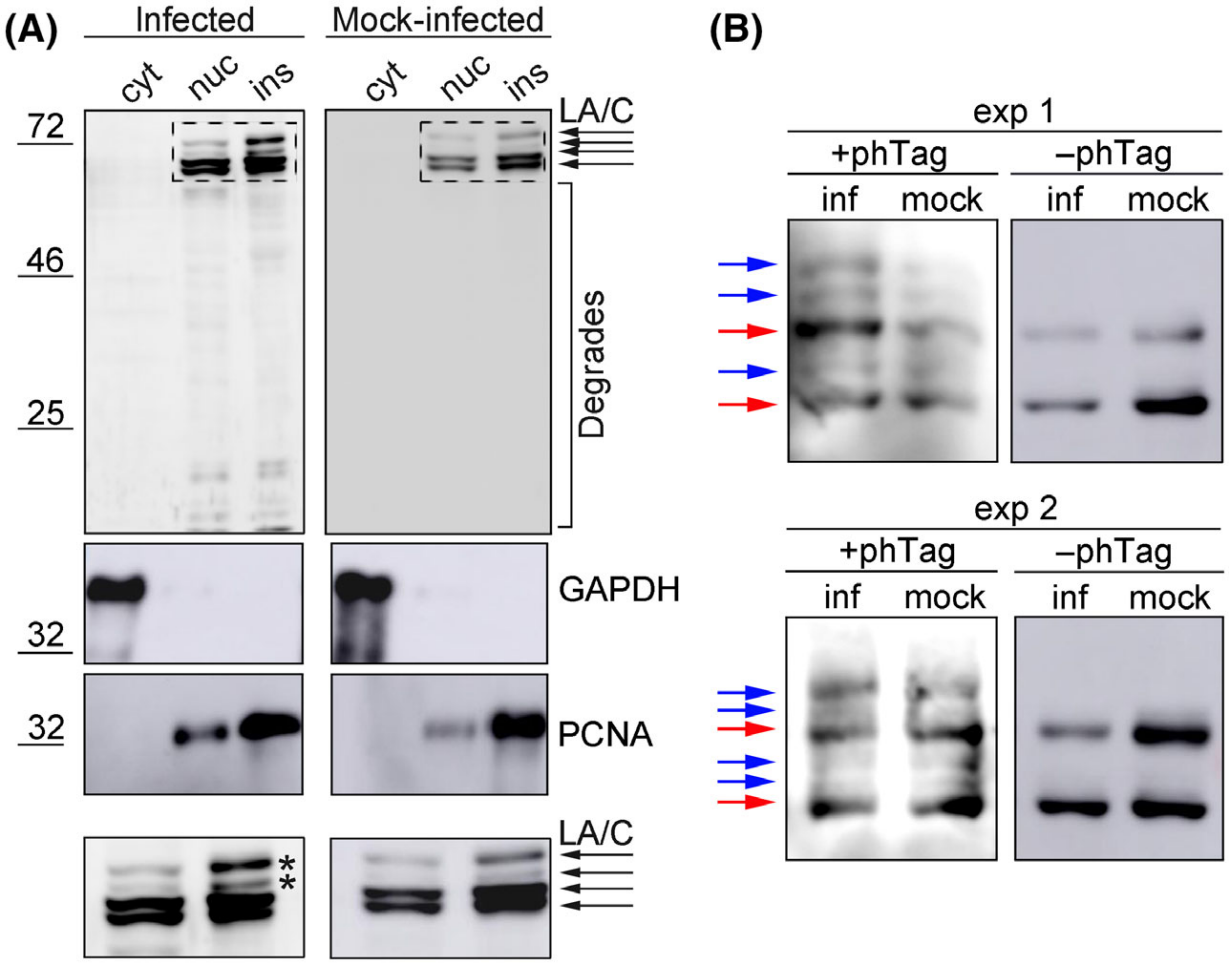
Lamin A/C is partially degraded and phosphorylated in infected cells. (A) Mouse 3T6 cells were infected with MPyV for 40 h, then fractionated into cytoplasmic (cyt), nuclear (nuc) and insoluble (ins) fractions. The same proportion of each fraction was separated by SDS/PAGE, transferred to a nitrocellulose membrane. Lamin A/C (L A/C) and as a control, GAPDH or proliferating cell nuclear antigen (PCNA) were detected using a specific antibody. Enlarged details of the western blot marked using a dashed box are presented in the lower panels. A lamin A/C‐positive band found predominantly in the infected cells is marked by an asterisk (*). (B) Mouse 3T6 cells were infected with MPyV and lysed at 40 h post‐infection. Lysates were separated by 6% SDS/PAGE gel supplemented with Phos‐tag (+phTag) or by control 6% SDS/PAGE gel (−phTag). Separated proteins were transferred to a nitrocellulose membrane and lamin A/C was detected with a specific antibody. Lamin A/C is marked by a red arrow and phosphorylated lamin A/C isoforms are marked by blue arrows.

We next investigated whether the increased solubility of lamin A/C resulted in the relocalisation of the protein to the cytoplasm and its subsequent degradation. Infected and mock‐infected cells were fractionated and the presence of lamin A/C in cytoplasmic, nuclear and insoluble, residual fraction was examined by western blotting. In both cell types (infected and mock‐infected), lamin A/C was detected in nuclear and insoluble fractions but no lamin A/C signal was detected in the cytoplasmic fraction (Fig. 9A). Several lamin A/C isoforms were found and some of them were more abundant in infected lysates. In addition, in contrast to mock‐infected cells, minute amounts of degraded lamin A/C were detected in the lysates of infected cells.

Alterations in protein solubility and altered protein migration in gel electrophoresis are frequently caused by post‐translational modifications of proteins. In the case of A‐type lamins, the post‐translational modification considered responsible for such observed effects is phosphorylation [22]. To investigate the phosphorylation status of lamin A/C in MPyV‐infected cells, the protein lysates were separated in SDS/PAGE gels supplemented with Phos‐tag (FUJIFILM Wako Pure Chemical, Osaka, Japan). This tag is a functional molecule that captures phosphorylated Ser/Thr/Tyr and His/Asp/Lys in the presence of Mn^2+^ ions. Thus, phosphorylated proteins are trapped by the tag present in the gel and individual isoforms can be separated by the amount and position of phosphorylation. Mouse fibroblasts were infected with MPyV for 40 h, lysates were separated in SDS/PAGE gels supplemented with Phos‐tag, and lamin A/C isoforms were detected by western blotting. In the control gel without Phos‐tag, two bands corresponding to lamin A and lamin C were detected in infected and control lysates (Fig. 9B). Two prominent bands with similar mobility were also detected in lysates separated in gels containing Phos‐tag. In addition to these bands, several other bands with slower migration were detected in infected and control lysates. Moreover, two bands with the highest molecular weight were more abundant in infected lysates (Fig. 9B) suggesting that there are different phosphorylation patterns or hyperphosphorylation of lamin A/C in infected cells compared with non‐infected cells.

### Absence of lamin A/C affects VP1 positioning in the cell nucleus but it is expendable for its association with the nuclear lamina

**Fig. 10:**
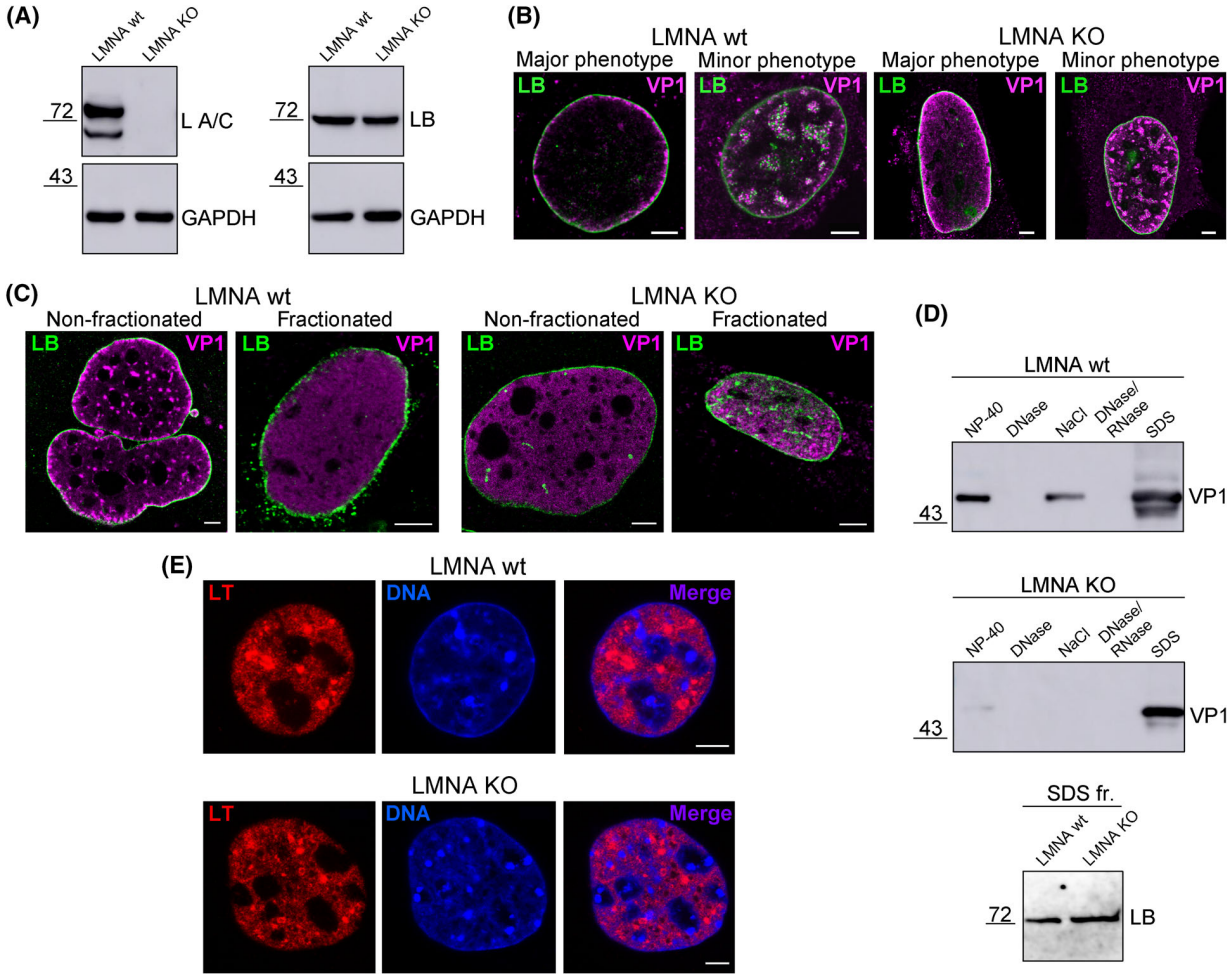
Lamin A/C deficiency does not influence the association of VP1 with the nuclear lamina but negatively affects VP1 positioning in the nucleus. (A) Lysates of LMNA KO cells and LMNA wild‐type (wt) cells were separated by SDS/PAGE and transferred to a nitrocellulose membrane, and lamin A/C (L A/C), lamin B1 (LB) and GAPDH were detected with specific antibodies. (B) LMNA KO cells and LMNA wt cells were infected, and at 40 h post‐infection (hpi), lamin B1 (green) and VP1 (magenta) were stained using specific antibodies. Images are confocal sections of the indicated cells. Bar, 5 μm. (C) LMNA KO cells and LMNA wt cells were infected, fractionated *in situ* at 40 hpi and VP1 (magenta) and lamin B1 (LB; green) were stained using specific antibodies. Images are selected confocal sections of non‐fractionated cells or cells after the final fractionation step. Bar, 5 μm. (D) LMNA KO cells and LMNA wt cells were infected, fractionated *in situ* at 40 hpi and washed‐out material from each fraction was separated by SDS/PAGE and transferred onto a membrane. The presence of VP1 in each washed‐out fraction and the presence of lamin B1 (LB) in the last (SDS) fraction was determined using specific antibodies. (E) LMNA KO cells and LMNA wt cells were infected, and at 40 hpi, LT (red) was stained with specific antibodies. DNA (blue) was stained with DAPI. Images are confocal sections of the indicated cells. Bar, 5 μm.

Our data indicated that VP1 accumulates in close proximity to the nuclear lamina. Therefore, we wondered whether VP1 nuclear localisation and its association with the nuclear lamina was affected in LMNA KO cells, which lack the *LMNA* gene and thus do not express A‐type lamins [23]. The absence of lamin A/C expression in LMNA KO cells was confirmed by western blotting of cell lysates using an antibody specific to lamin A/C (Fig. 10A). LMNA KO cells and their wild‐type counterpart (LMNA wt cells) were infected with MPyV for 40 h, then VP1 and lamin B1 were stained using specific antibodies and the cells were analysed by confocal microscopy. In both cell types, two VP1 staining patterns were detected (Fig. 10B). In most LMNA wt cells, VP1 was associated with the nuclear lamina but the diffuse nuclear staining pattern was detected in most LMNA KO cells. Moreover, in some LMNA wt or KO cells, VP1 formed speckles inside the nucleus and these spots were juxtaposed with the intranuclear lamin B1 signal (Fig. 10B).

To verify that the presence of lamin B1 alone is sufficient for VP1 association with the nuclear lamina, *in situ* fractionation of LMNA wt and KO cells were performed, and residual structures were analysed by fluorescence microscopy. After fractionation, VP1 was detected throughout the nucleus area with occasional colocalisation of VP1 with lamin B1 signals in both cell types (Fig. 10C). Concurrently, the analysis of wash‐out material showed that VP1 was predominantly found with lamin B1 in the last insoluble SDS fraction in both cell types (Fig. 10D). In addition, VP1 was detected in almost all fractions in wt cells. In contrast, only a weak VP1 signal was detected in the soluble NP‐40 fraction in LMNA KO cells (Fig. 10D). Although the absence of lamin A/C did not affect the association of VP1 with the nuclear lamina, the absence did negatively influence subnuclear VP1 localisation, indicating the possible participation of lamin A/C in the intranuclear movement of VP1 or newly synthetised virions.

To verify whether the lack of lamin A/C affects the formation of VRC, LT antigen was stained by specific antibodies at 40 hpi in LMNA wt and KO cells. No difference in VRC formation was detected between LMNA KO cells and LMNA wt cells (Fig. 10E). In both cell types, LT antigen was found in distinct spots apparently representing VRCs. Thus, an absence of lamin A/C does not appear to affect VRC formation.

### Lamin A/C deficiency in cells hampers the MPyV replication cycle

**Fig. 11:**
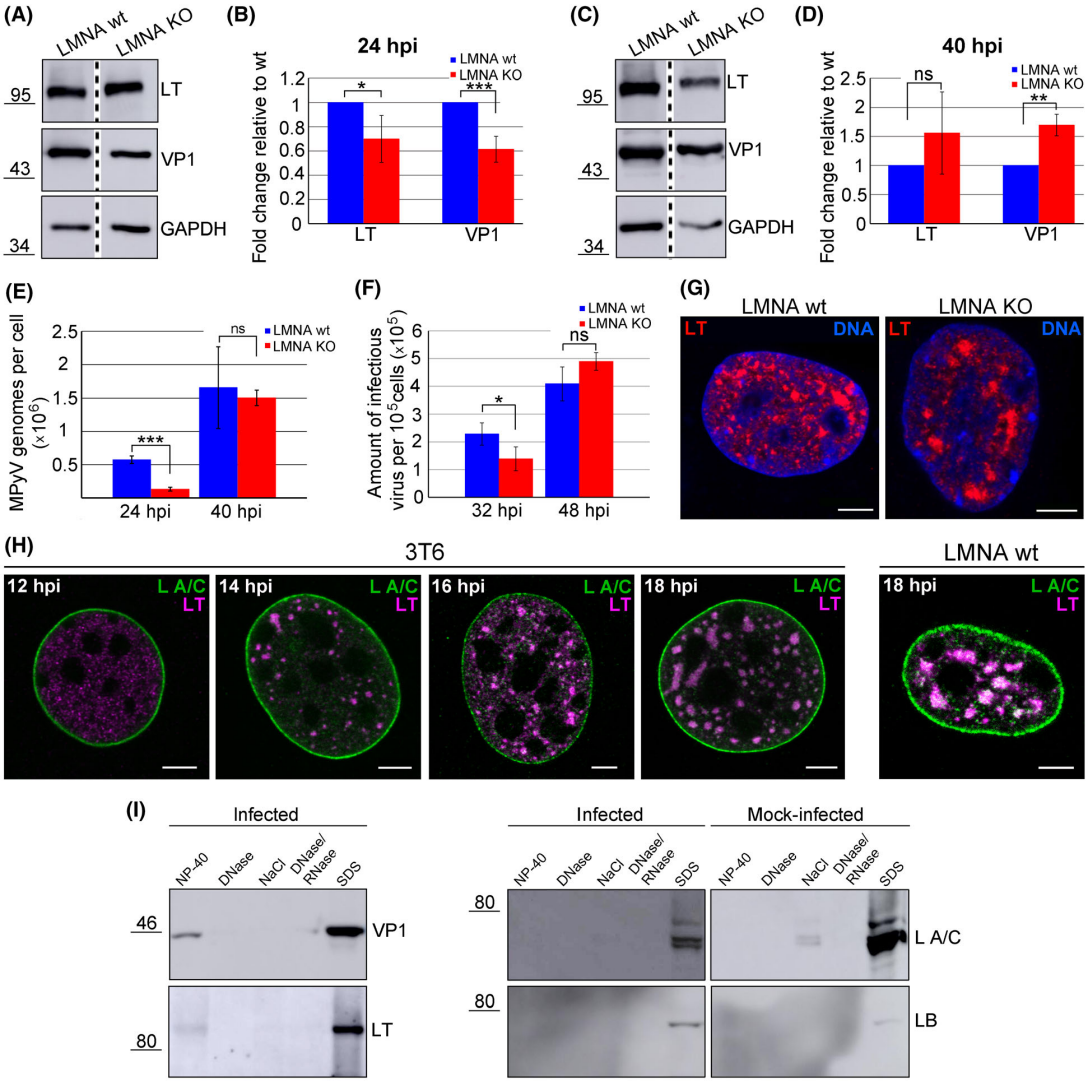
A lack of lamin A/C slows the virus replication cycle. (A, C) LMNA KO cells and LMNA wt cells were infected, then lysed at 24 (A) or 40 (C) h post‐infection (hpi). Lysates were separated by SDS/PAGE, transferred onto a nitrocellulose membrane, and LT, VP1 and GAPDH were detected using specific antibodies. Western blots were spliced as indicated by the dashed vertical lines. (B, D) Graphic illustration of densitometry analysis of the digital images of western blots from four independent experiments. The data show the fold increase relative to LMNA KO cells. Error bars represent the standard deviation (SD). Student's *t*‐test was used for statistical analyses. *P < 0.05; **P < 0.01 and ***P < 0.001; ns, not significant. (E) LMNA KO cells and LMNA wt cells were infected and the number of MPyV genomes in cells was measured by quantitative PCR at 24 and 40 hpi. Data are the mean from triplicates of one experiment ± SD. Student's *t*‐test was used for statistical analyses. ***P < 0.001; ns, not significant. One of two representative experiments is displayed. (F) LMNA KO cells and LMNA wt cells were infected, and at 32 or 48 hpi, MPyV virions were isolated. Mouse 3T6 cells were infected with equal volumes of the virus isolated from the LMNA KO or LMNA wt cells. Cells were fixed at 24 hpi, stained using an LT‐specific antibody and virus titres were assigned. The graph shows the mean values from three independent experiments ± SD. Student's *t*‐test was used for statistical analyses. *P < 0.05; ns, not significant. (G) LMNA KO cells and LMNA wt cells were infected, and at 18 hpi, LT (red) was stained using specific antibodies. DNA (blue) was stained with DAPI. Images are confocal sections of the indicated cells. Bar, 5 μm. (H) LMNA wt cells and 3T6 cells were infected and lamin A/C (L A/C; green) and LT (magenta) were stained with specific antibodies at the indicated hours post‐infection. Only 18 hpi is shown for LMNA wt cells. Images are confocal sections of the indicated cells. Bar, 5 μm. (I) Mouse 3T6 cells were infected, fractionated *in situ* at 24 hpi and washed‐out material from each fraction was separated by SDS/PAGE and transferred onto a nitrocellulose membrane. The presence of VP1, LT, lamin A/C and lamin B1 (LB) in each washed‐out fraction was determined using specific antibodies.

Based on our finding that the absence of lamin A/C affects VP1 localisation within the nucleus, we wondered whether the lack of lamin A/C induced other changes in the virus replication cycle. Thus, LMNA wt and LMNA KO cells were infected, lysed at 24 or 40 hpi and the amount of VP1 and LT was analysed by western blotting. Compared with wt cells, LT and VP1 levels decreased to 70% and 61%, respectively, in LMNA KO cells at 24 hpi. However, at 40 hpi, the amount of LT increased by 56% and the amount of VP1 increased by 70% (Fig. 11A–D). As LT antigen is indispensable for virus propagation, the different dynamics of LT production in LMNA KO cells may impact virus replication. To investigate virus replication, the number of genomes per cell was assessed using quantitative PCR (qPCR). Only a moderate increase in the amount of viral DNA between the 24 and 40 hpi was detected. Since MPyV virus isolations showed a high ratio of the total number of particles to infectious particles (usually 1 infectious particle per 10 000 total virus particles), the replicating genomes at 24 hpi might be probably masked by persisting ‘input’ genomes which in turn may decrease the 24:40 hpi ratio. Nevertheless, at 24 hpi, the number of genomes significantly decreased to 23% in LMNA KO cells in comparison with wt cells, whereas at 40 hpi, the number of genomes in LMNA KO cells was almost equal to that in LMNA wt cells (Fig. 11E). A similar trend was observed when the amount of infectious virus was analysed. The amount of virus declined to 60% in LMNA KO cells at 32 hpi, but later (48 hpi), a slightly increased (by 20%) virus amount was detected (Fig. 11F). These data suggest that a lack of lamin A/C negatively affects the early stages of infection. Replication of MPyV DNA starts at 16–18 hpi [24]. We expected that if lamin A/C participates in VRC formation, in the absence of lamin A/C, the LT antigen signal would be more diffuse. However, at 18 hpi, distinct spots of LT – representing VRCs – were observed in LMNA KO cells and in wt cells (Fig. 11G). This finding indicates that the absence of lamin A/C does not affect the formation of VRCs at early times post‐infection. However, apart from mechanical functions, lamin A/C is also involved in other processes, such as gene regulation [25]. To verify whether lamin A/C is a part of VRCs in the early stages of infection, colocalisation of lamin A/C and LT was examined at the 12–18 hpi interval in 3T6 and LMNA wt cells. In both cell types, the intranuclear lamin A/C signal significantly colocalised with the LT signal as soon as VRCs were detectable in the nucleus (from 14 hpi) (Fig. 11H), demonstrating that lamin A/C is part of the VRCs at early times post‐infection. Finally, the integrity of the nuclear lamina was tested at early times post‐infection. No disruption of lamin A/C staining was detected at early times post‐infection (12–32 hpi) (Fig. 11H, Fig. 2). Simultaneously, at 24 hpi, no changes in the solubility of either lamin A/C or lamin B1 were detected in infected cells compared to mock‐infected cells (Fig. 11I).

Taken together, these data suggest that lamin A/C is crucial in the early stages of infection. Significant morphological changes of the lamina – detected only at 40 hpi – are most likely only associated with virus egress from the nucleus and are not required for the regulation functions of lamin A/C in the early stages of infection. Although the absence of lamin A/C does not affect the formation of VRCs, it negatively influences virus gene expression and genome replication, which results in the deceleration of the virus replication cycle in the early phases of infection.

## Discussion

The nuclear lamina is an eminent barrier that must be surpassed by nuclear‐replicating viruses for their successful propagation. Different virus families have evolved distinct strategies to circumvent this obstacle. The mechanisms to overcome the nuclear lamina are best characterised by viruses from the family *Herpesviridae*. In the early phases of infection, nuclear pores are used for genome delivery to the nucleus. The viral capsid is transported along microtubules to the centrosome and further to the nuclear pore where the capsid docks, then the viral genome is transported to the nucleus through the nuclear pore complex [26]. At later times post‐infection, phosphorylation of lamin A/C mediated by virus‐encoded kinases (β‐ and γ‐herpesviruses) or cellular kinases (α‐herpesviruses) leads to disintegration of the nuclear lamina, facilitating subsequent capsid escape from the nucleus [16].

In contrast, information about virion egress from cells infected with polyomaviruses is limited. The closely related polyomaviruses JCPyV and BK polyomavirus (BKPyV) use agnoprotein, which is a late non‐structural protein, for virus release from the nucleus [17, 27]. However, the effect of agnoprotein on the nuclear lamina remains elusive. In cells expressing JCPyV agnoprotein, increased lateral mobility of the lamin B receptor, which is the integral protein of the inner nuclear lamina, was detected [17]. However, in BKPyV‐infected cells, lamin B localisation was not affected [27], suggesting that gross destabilisation of the lamina does not occur in polyomavirus‐infected cells. In MPyV‐infected cells, we detected accumulation of the major capsid protein, VP1, under the nuclear lamina at late times post‐infection. This signal apparently corresponds to previously detected virions at the nuclear periphery [28]. In infected cells, depletion of lamina staining was observed and lamin A/C itself was affected, including detection of a decreased level of lamin A/C, modest lamin A/C degradation and different phosphorylation patterns compared with uninfected cells. These findings support the hypothesis that the nuclear lamina is disrupted in MPyV‐infected cells which facilitates non‐lytic virus egress in the late phase of infection. However, no disruptions in nuclear permeability were observed in the late phase of infection. As the infection proceeds, progeny virions accumulate under the lamina and within the nucleus, which increases the volume of the nucleus and causes expansion of the nuclear lamina meshwork [29]. In infected cells, we found increased levels of lamin B1, but decreased levels of lamin A/C. Nevertheless, we hypothesised that the nuclear lamina in infected cells is thinner than in uninfected cells, allowing virion progeny to egress through larger gaps between filaments. However, in LMNA KO cells, we did not detect an increased signal of VP1 protein in the cytoplasm of infected cells, suggesting that the absence of lamin A/C did not facilitate the nuclear egress of the virus. The structure and mechanics of the lamin meshwork are strictly dependent on each other [30, 31]. An absence of lamin A/C leads to the enlargement of the area between lamin B1 fibres [30] and changes in the mechanical properties of the nuclear lamina [31]. Infection had a marked effect on the lamin A/C meshwork, potentially leading to its limited and/or local disintegration. In infected cells, we detected changes in lamin A/C phosphorylation. Lamin A/C phosphorylation is linked to with dissociation of lamin A/C filaments from the lamin meshwork [22]. Collectively, these changes may contribute to limited and local dissociation of the nuclear lamina, thus enlarging the space between individual filaments. The increased pressure of virion progeny on the lamina in the late phase of infection may promote virion egress from the infected nucleus as the less‐interconnected meshwork of the nuclear lamina is unable to withstand greater forces compared with a highly interconnected meshwork [31]. In the final steps of viral infection, virion egress may be facilitated by caspase‐dependent cleavage of lamins [32] as caspases are activated in the very late phases of infection [33]. Although MPyV does not encode agnoprotein, the minor structural proteins, VP2 and VP3, may partially compensate for agnoprotein role in virus egress. Membrane affinity [33] and viroporin activity [10] have been demonstrated for VP2 and VP3, therefore the involvement of these structural proteins in virion egress from the nucleus can be presumed.

Nuclear lamins may not only be restriction factors for virus infections. In addition to mechanical functions, nuclear lamins participate in chromatin organisation and gene expression. We detected lamin–MPyV DNA complexes containing LT antigen, which is a viral protein essential for genome replication. This suggests that the lamina may function as a scaffold for VRCs. We also detected complexes of VP1 associated with nuclear lamins. Our results suggest that complexes of viral and nuclear proteins are associated with lamin A/C and lamin B1 meshwork. Both types of lamins may interact with DNA at chromatin regions termed lamin‐associated domains (LADs). LADs are involved in organising the genome architecture within the nucleus (reviewed in [21]). We initially thought to perform lamin A/C or lamin B1 knock‐down experiments to characterise their role in MPyV infection. Unfortunately, we were not able to obtain conclusive results probably because of the long half‐life of lamins. Due to the kind provision of the lamin A/C knock‐out cell line [23], we could further focus on the role of lamin A/C in the MPyV replication cycle. We observed that an absence of lamin A/C affected localisation of VP1 within the nucleus. A‐type lamins are localised at the nuclear periphery and in the nucleoplasm where they may interact with chromatin and regulate gene expression and chromatin mobility (reviewed in ref. [21]). In LMNA KO cells infected with herpes simplex 1 virus, tethering of the replication compartments at the nuclear periphery was disrupted, indicating that loss of lamin A/C means viral genomes are unable to target the nuclear periphery and develop replication compartments [34]. Lamin A/C may also be involved in the movement of VP1–genome complexes toward the nuclear periphery. Apart from the virus genome itself, the pleiotropic transcription regulator YY1 may participate in targeting the VP1–genome complexes to the nuclear periphery. YY1 belongs to the Polycomb group proteins and is involved in transcriptional repression [35]. In addition, YY1 participates in chromatin targeting the nuclear lamina, as the knockdown of lamin A/C or YY1 abolished association with chromatin [36]. YY1 binds to the MPyV genome [37, 38] and also directly interacts with VP1 [39]. Therefore, YY1 may mediate the interaction of VP1–genome complexes with the nuclear lamina.

A lack of lamin A/C did not prevent the formation of mouse polyomavirus VRCs, suggesting that lamin A/C does not have a structural function in VRC formation. However, in LMNA KO cells, viral gene expression and genome replication were affected. Therefore, lamin A/C has a regulatory, rather than structural, role in MPyV replication. Lamin A/C may be involved in several steps of virus replication, including genome uncoating, transcription and/or regulation of genome replication. Initiation of transcription of early genes is supported by poly(ADP‐ribose) polymerase‐1 (PARP‐1). PARP‐1 removes VP1 from the MPyV enhancer, promoting the interaction with early transcription factors [40]. PARP‐1 has been reported to be associated with lamins [41]. Therefore, the absence of lamin A/C may lead to delayed or inefficient initiation of early transcription.

The absence of lamin A/C has a global impact on transcription. In the nuclear interior, A‐type lamins do not form higher‐order structures. However, there are two fractions of A‐type lamins in the nuclear interior of interphase nuclei: phosphorylated lamin A/C or non‐phosphorylated lamin A/C in complex with LAP2α [42]. Although phosphorylated lamin is a hallmark of mitosis, lamin phosphorylated at Ser22 and Ser392 is also found in interphase nuclei [22]. A‐type lamins phosphorylated at Ser22 were found to interact with genomic sites containing features of active enhancers, near genes undergoing active replication [43]. Phosphorylated lamin C binding sites overlap with genomic sites occupied by the AP‐1 transcription factor [43]. In addition, disruption of lamin A/C deregulates AP‐1 activity, constitutively activating AP‐1‐dependent DNA‐binding and transcriptional activity [44]. AP‐1, which is a heterodimer of Fos and Jun, binds to MPyV transcription and replication enhancers and promotes virus transcription and replication [45, 46]. Our findings of the increased solubility of lamin A/C and its altered phosphorylation pattern in the late phase of infection, coupled with the altered amounts of LT and VP1 proteins observed in the early and late phases of infection in LMNA KO cells, suggest that A‐type lamins are involved in regulating MPyV gene expression.

Finally, lamins are instrumental in DNA replication. We detected an association of VRCs with lamin A/C and lamin B1. In addition, our observation of reduced MPyV genome replication and co‐localisation of LT antigen with lamin A/C in the early stages of infection suggest that lamins participate in MPyV genome replication. VRCs were found adjacent to another nuclear structure, promyelocytic nuclear bodies [47], which have been shown to affect viral genome expression and/or replication of several DNA viruses [48]. However, knocking out the main component of these nuclear bodies, the PML protein, did not affect VRC formation or virus replication, thus the role of the promyelocytic nuclear bodies in MPyV replication remains to be elucidated [47, 48]. Several other cellular proteins involved in DNA replication and the DNA damage response (DDR) have been shown to be associated with VRCs [47, 49]. These cellular proteins organise VRCs into two spatially and functionally distinct subdomains [50]. LT localises to the replication‐associated subdomain, whereas DDR signalling proteins, such as phosphorylated ataxia‐telangiectasia mutated kinase or a subunit of replication protein A, RPA32, localise to the repair‐associated subdomain. Both types of lamins are involved in DNA replication and may be associated with different subdomains of VRCs. Lamin B1 co‐localises with proliferating cell nuclear antigen (PCNA) in replication factories [51] and is crucial for the elongation of newly synthetised DNA, thus is probably localised to the replication‐associated subdomain. In contrast, A‐type lamins are likely important for the establishment of early S‐phase replication sites [52]. Presumably, an absence of lamin A/C does not lead to aberrant MPyV genome replication, but rather delays initiation of the process. Lamin A/C can recruit ssDNA binding proteins (replication protein A) to replication forks thus protecting them against nuclease degradation [53]. Similarly, loss of lamin A/C results in delayed replication not termination. A significant defect in the recruitment of DDR proteins (Mre11, CtIP or replication protein A) to DNA damage foci was observed in lamin A/C‐deficient cells [54], suggesting that lamin A/C may be part of both subdomains of VRCs.

In conclusion, our results suggest that the nuclear lamina and nuclear lamins play crucial but diverse role in the MPyV life cycle. Both types of lamins represent a physical barrier to the nuclear egress of MPyV. In addition, lamin B1 serves as a platform for the organisation of VRCs, whereas A‐type lamins participate in gene expression regulation, replication and potential movement within the nucleus. Further studies are needed for a detailed understanding of the contribution of lamins to MPyV replication.

## Materials and methods

### Cell lines and viral infection

Mouse fibroblasts 3T6 (ATCC; CCL‐96), murine mammary gland cell line (NMuMG) (ATCC; CRL‐1636), mouse fibroblasts NIH‐3T3 (ATCC; CRL‐1658), WOP cells stably expressing LT antigen [55], LMNA KO cells [23], mouse fibroblasts with *LMNA* knock out and their wild‐type counterpart – LMNA wt cells – were grown at 37 °C in a 5% CO_2_–air humidified incubator using Dulbecco's Modified Eagle's Medium (DMEM; Merck, Rahway, NJ, USA) supplemented with 10% bovine serum (Thermo Fisher Scientific, Waltham, MA, USA). Mouse polyomavirus strain BG (GenBank: J02289.1) was isolated and purified from infected 3T6 cells using a 10% (w/w) sucrose cushion according to a standard protocol [56]. Before infection, cells were synchronised by starvation for 24 h [24]. For infection, cells were incubated for 1 h with a virus inoculum at a multiplicity of infection (MOI) of 1–10 plaque‐forming units (pfu) per cell, depending on the experimental settings.

### Antibodies

The primary antibodies used in the study were mouse monoclonal antibody to VP1 [57], rabbit polyclonal antibody to VP1 (prepared in our laboratory), rat monoclonal antibody to LT [58], rat monoclonal antibody against a common region of MPyV T antigens [58], goat polyclonal antibody to lamin B (Santa Cruz Biotechnology, Dallas, TX, USA), rabbit monoclonal antibody to lamin B1 (Abcam, Cambridge, UK), mouse monoclonal antibody to lamin A/C (Cell Signaling Technology, Danvers, MA, USA), rabbit polyclonal antibody to glyceraldehyde 3‐phosphate dehydrogenase (Merck), goat polyclonal antibody to proliferating cell nuclear antigen (Santa Cruz Biotechnology) and rabbit polyclonal antibody to lamin B receptor (Abcam). The secondary antibodies were goat anti‐mouse and goat anti‐rabbit antibodies conjugated with peroxidase (both from Bio‐Rad, Hercules, CA, USA), donkey anti‐goat conjugated with peroxidase (Santa Cruz Biotechnology), goat anti‐rat antibody conjugated with peroxidase (Cell Signaling Technology), donkey anti‐mouse antibody conjugated with Alexa Fluor‐488, donkey anti‐goat antibody conjugated with Alexa Fluor‐488, donkey anti‐rabbit antibody conjugated with Alexa Fluor‐488, donkey anti‐rabbit antibody conjugated with Alexa Fluor‐546, goat anti‐mouse antibody conjugated with Cy3, goat anti‐rat antibody conjugated with Cy3, goat anti‐rat antibody conjugated with Alexa Fluor‐488, goat anti‐rabbit antibody conjugated with Alexa Fluor‐633, goat anti‐rat antibody conjugated with Alexa Fluor‐647, chicken anti‐mouse antibody conjugated with Alexa Fluor‐647 and donkey anti‐rabbit antibody conjugated with Alexa Fluor‐594 (all from Thermo Fisher Scientific). The secondary antibodies used for STED microscopy were Abberior STAR 635P goat anti‐mouse IgG, Abberior STAR 580 goat anti‐rabbit IgG, Abberior STAR 635P donkey anti‐goat IgG and Abberior STAR 580 goat anti‐rat IgG (all from Abberior GmbH, Göttingen, Germany).

### Plasmids and cells transfection

Transfection of NIH‐3T3 or WOP cells was performed by electroporation in the Nucleofector™ device using Nucleofector V solution (Lonza, Basel, Switzerland) according to the manufacturer's instructions. Briefly, 4 × 10^6^ exponentially growing cells were mixed with 6 μg of pEF1‐LATE plasmid DNA producing MPyV structural proteins [59] and 100 μL of Nucleofector V solution and electroporated (programme U‐030).

### *In situ* fractionation

*In situ* fractionation was performed according to Staufenbiel and Deppert [19] and has been described previously in Horníková *et al*., 2017 and 2020 [20, 60]. Briefly, cells grown in 6‐well dishes were washed three times with KM buffer [10 mm MES, pH 6.2; 10 mm NaCl; 1.5 mm MgCl_2_; 10% glycerol; and a cocktail of protease inhibitors (Roche, Basel Switzerland)], followed by four consecutive extractions with KM buffer with different additives. The first extraction was performed with KM buffer supplemented with 1% NP‐40, 1 mm EGTA and 5 mm DTT. The buffer (0.2 mL) was added to the dish, incubated for 3 min on ice and removed and stored. Subsequently, another 0.5 mL of the buffer was added to the dish, incubated for 27 min on ice and then the extract was combined with the previous extract and marked as the NP‐40 fraction. The second extraction was performed with 0.25 mL of KM buffer supplemented with DNase I (100 U mL^−1^; Roche) for 15 min at 37 °C. The extract was removed and marked as the DNase fraction. The third extraction was performed with 0.25 mL of KM buffer supplemented with 2 m NaCl, 1 mm EGTA and 5 mm DTT for 30 min on ice. The extract was removed and marked as the NaCl fraction. The fourth extraction was performed with 0.375 mL of KM buffer supplemented with DNase I (100 U mL^−1^; Roche) and RNase A (5 U mL^−1^; Serva Electrophoresis GmbH, Heidelberg, Germany). The extract was removed and marked as the DNase/RNase fraction. The remaining highly insoluble structures were dissolved in a KM buffer containing 1% SDS and marked as the SDS fraction. After each extraction, the structures on the dish were washed three times with KM buffer. Proteins in the extracted fractions were precipitated with acetone, dissolved in Laemmli buffer and analysed by SDS/PAGE. For light microscopy studies, cells were seeded onto coverslips and fractionated as above (the amounts of solutions were adapted accordingly). Structures remaining on the coverslip after each fractionation step were fixed and labelled as described hereafter.

### Immunofluorescence staining

Coverslips with intact cells or structures from cells after *in situ* fractionation were washed in phosphate‐buffered saline (PBS; Lonza) or KM buffer, respectively. At the indicated time points, the cells were fixed in 3.7% paraformaldehyde (PFA) in PBS for 15 min, permeabilised by 0.5% Triton X‐100 in PBS for 5 min and washed in PBS three times. The samples were then blocked with 0.25% gelatin and 0.25% bovine serum albumin in PBS for 30 min. Immunostaining with primary and secondary antibodies was conducted for 1 h and 30 min, respectively. After the antibody incubations, the samples were extensively washed (3 × 10 min) with PBS. Finally, the samples were incubated for 3 min in a solution containing Hoechst stain (10 μg mL^−1^) in deionised water, washed in deionised water and mounted on droplets of Antifade Fluorescence Mounted Medium (Abberior GmbH). Images were obtained using a Zeiss LSM 880NLO confocal microscope (Carl Zeiss, Oberkochen, Germany) or by STED super‐resolution microscopy using a confocal microscope Abberior Instruments STED.

### Image data analysis after STED super‐resolution microscopy

Before the analysis, STED and confocal images were deconvolved in huygens software (https://svi.nl/Huygens-Professional) using the default deconvolution settings and the specified microscope parameters. The Pearson correlation coefficient was then computed using the following formula that was directly implemented in Python.

$$r_{\mathrm{xy}}=\frac{\sum i=1^{n}\left(x_{i}-\bar{x}\right)\left(y_{i}-\bar{y}\right)}{\sqrt{\sum i=1^{n}{\left(x_{i}-\bar{x}\right)}^{2}}\sqrt{\sum i=1^{n}{\left(y_{i}-\bar{y}\right)}^{2}}}$$

where $x_{i}$ and $y_{i}$ define i‐th pixels of different channels.

Furthermore, we aimed to better describe spatial relationships between lamin and VP1. To compute the distances, lamin and VP1 were localised. First, the lamin protein signal associated with the nuclear envelope was segmented. The mask for lamin was then manually edited to exclude the signal from the centre of the nucleus. Next, the VP1 signal was detected using the Laplacian of Gaussian filter implemented in the scikit‐image [61] Python package with the following parameters: max_sigma = 5, num_sigma = 10, threshold = 0.015. Once lamin and VP1 were localised, the Euclidian distance for each VP1 to the closest lamin signal pixel was computed. All analyses were performed in a custom Python script.

### Live cell assay for nuclear envelope disruption

Live NMuMG or 3T6 cells infected with MPyV (MOI = 10 pfu/cell) or mock‐infected, growing on glass‐bottom dishes (MatTek, Ashland, MA, USA) for 40 hpi, were washed in PB buffer [20 mm HEPES‐KOH (pH 7.4), 110 mm potassium acetate, 5 mm magnesium acetate, 0.5 mm EGTA and 250 mm sucrose], permeabilised in PB containing 15 μg mL^−1^ digitonin for 5 min, and then washed again three times in PB. Permeabilised cells were incubated for 1 min in TB buffer [20 mm HEPES‐KOH (pH 7.3), 110 mm potassium acetate, 5 mm sodium acetate, 1 mm EGTA and 2 mm dithiothreitol] containing 155‐kDa TRITC‐labelled dextran (250 μg mL^−1^) (Merck). The live cells were visualised immediately using a Zeiss LSM 880NLO confocal microscope (Carl Zeiss) equipped with an Okolab Microscope Incubator and Gas Controller maintaining the temperature (37 °C) and 5% concentration of CO_2_. Cell images were captured at 3–40 min intervals, the last time being 40 min after the addition of labelled dextran.

### Combined immunofluorescence and fluorescence *in situ* hybridisation (FISH)

FISH was performed according to the protocol described by Solovei and Cremer [62] with minor modifications. Briefly, cells grown on glass coverslips were infected with MPyV (MOI = 5 pfu per cell), fixed at 40 hpi, permeabilised and indirect immunofluorescence staining of proteins was performed as described above. Subsequently, cells were post‐fixed with 2% PFA in PBS for 10 min and washed in PBS (3 × 5 min). Samples were then treated with 50 μg mL^−1^ RNase A in PBS at 37 °C for 1 h, washed in PBS (3 × 5 min) and incubated for 1 h in 20% glycerol in PBS. Cells were permeabilised for a second time using three freeze–thaw cycles in liquid nitrogen. Next, samples were treated with 0.1 m HCl for 5 min, washed in PBS (2 × 5 min), equilibrated in 2× SSC (0.3 m NaCl, 0.03 m sodium citrate) for 5 min and incubated overnight in 50% formamide in 2× SSC buffer. A biotin‐labelled probe was prepared by the BioNick DNA Labeling System (Thermo Fisher Scientific) according to the manufacturer's protocol using pMJG plasmid [28] as the template. The probe was precipitated together with unlabelled salmon sperm DNA (Thermo Fisher Scientific) and diluted in hybridisation solution (50% formamide and 10% dextran sulfate in 2× SSC) to achieve a probe concentration of 10 ng μL^−1^. The hybridisation mixture was then applied to the cells and simultaneously denatured for 5 min at 80 °C. Hybridisation was performed overnight at 37 °C. Non‐specifically bound and excess probe was removed by three 10‐min washes in 2× SSC at 37 °C and three 5‐min washes in 0.1× SSC at 60 °C. Detection of the hybridised, biotin‐labelled probe was achieved using streptavidin conjugated with Alexa Fluor 594. Cells were mounted on Abberior Mount Liquid Antifade (Abberior), and images were obtained using a Zeiss LSM 880NLO confocal microscope (Carl Zeiss).

### Western blot analysis

The method has been described previously in Horníková *et al*. [60]. Cells were harvested at the indicated time points after infection or transfection, then were washed with PBS and lysed in ice‐cold RIPA buffer [150 mm NaCl, 5 mm EDTA, 50 mm Tris–HCl (pH 7.4), 0.05% NP‐40, 1% sodium deoxycholate and 1% Triton X‐100, 0.1% SDS] supplemented with a cocktail of protease inhibitors (Roche) for 20 min on ice. Cell debris was removed by centrifugation (20 000 ***g***, 30 min, 4 °C). The protein samples were resolved by 10% SDS/PAGE and electro‐transferred onto a nitrocellulose membrane in a blotting buffer (0.3% Tris, 1.44% glycine and 20% methanol) at 2.5 mA cm^−2^ for 90 min. The membranes were blocked in 5% non‐fat milk in PBS for 45 min. Immunostaining with primary and secondary antibodies was conducted for 1 h and 30 min, respectively. Incubation with each antibody was followed by extensive washing of the membrane in PBS. The membranes were developed using an enhanced chemiluminescence reagent (Thermo Fisher Scientific) and the signal was visualised by an Amersham Imager 600 RGB (GE Healthcare, Chicago, IL, USA). If necessary, the membrane was re‐probed according to Senepin *et al*. [63]. Briefly, the membrane was washed in PBS and the chemiluminescence signal was quenched by incubation in 30% peroxide for 15 min at 37 °C. Next, the membrane was washed for 15 min in water, then incubated for 15 min in PBS and 45 min in 5% non‐fat milk in PBS. Subsequently the membrane was re‐stained with antibodies. The band intensities of proteins were assessed using the Amersham Imager 600 RGB software and, if not stated otherwise, normalised to GAPDH levels. The fold change in the protein level was relativised to the control cells. Although the analyses of most western blots were performed using non‐cropped blots, some of the blots were cropped for clear presentation purposes.

### Cell fractionation

The 3T6 cells were infected with MPyV (MOI = 10 pfu per cell), harvested at 40 hpi and washed with PBS. Then, 2 × 10^5^ cells were fractionated to cytoplasmic, nuclear and insoluble fractions using a Nuclear & Cytoplasmic Extraction Kit (G‐Biosciences, Saint Louis, MO, USA) according to the manufacturer's protocol. Proteins from each fraction were separated by 10% SDS/PAGE, transferred onto a membrane and lamin A/C protein was detected with a specific antibody.

### Phosphorylation of lamin A/C in infected cells

Synchronised 3 × 10^5^ 3T6 cells were infected (MOI = 10 pfu per cell) and lysed at 40 hpi. The lysis buffer [50 mm Tris–HCl (pH 7.4), 70 mm β‐mercaptoethanol and 0.5% SDS] was pre‐boiled for 10 min and 100 μL were added to the PBS‐washed cells. Cells were scraped into the lysis buffer and the cell suspension was homogenised by passing through a needle (21G) 20 times. The suspension was then incubated for 10 min at 100 °C and four times diluted with 50 mm Tris–HCl (pH 7.4). Cellular debris was removed by centrifugation (20 000 ***g***, 20 min, 4 °C) and supernatants were resolved in a 6% SDS/PAGE gel supplemented with 20 μm Phos‐tag (FUJIFILM Wako Pure Chemical) and 100 μm MnCl_2_. The gel containing separated proteins was incubated in blotting buffer (0.3% Tris, 1.44% glycine, 0.1% SDS and 20% methanol) with constant agitation for 30 min and proteins were electro‐transferred onto a nitrocellulose membrane in a blotting buffer at 2.5 mA cm^−2^ for 90 min. The membranes were blocked in 5% non‐fat milk in PBS for 45 min and staining of proteins was performed as described above.

### Impact of LMNA knock out on viral protein levels and virus production

The method has been described previously in Horníková *et al*. [60]. LMNA KO cells or LMNA wt cells were infected (MOI = 10 pfu per cell) and lysed at 24 or 40 hpi. Lysates were resolved by 10% SDS/PAGE and transferred to a nitrocellulose membrane, and virus proteins were detected using specific antibodies. The band intensities of proteins were determined using Amersham Imager 600 RGB software and normalised to GAPDH levels. The fold increase of protein levels was compared with their levels in LMNA wt cells. To assess virus production, cells were lysed at 32 or 48 hpi in culture media using three freeze–thaw cycles. Cellular debris was removed by centrifugation (8000 ***g***, 10 min, 4 °C) and the supernatant was stored at 4 °C. Cellular debris was resuspended in 10 mm Tris–HCl (pH 7.4) supplemented with neuraminidase (0.01 U mL^−1^) and aprotinin (2 μg mL^−1^) and incubated with constant agitation overnight at room temperature. Next, the suspension was centrifuged (8000 ***g***, 10 min, 4 °C) and the supernatant was combined with the stored supernatant and used as virus inoculum in the following experiment. Mouse fibroblast 3T6 cells were infected with 0.01 volume of the virus inoculum, then were fixed at 24 hpi and LT antigen was stained using specific antibodies. The number of infected cells were scored by immunofluorescence microscopy. At least 300 cells were counted per experiment, and the amount of infectious viral particles in the viral inoculum was assessed. The amount of virus produced in LMNA KO cells was compared with that produced in LMNA wt cells.

### Impact of LMNA knock out on viral genome replication

LMNA KO or LMNA wt cells were infected with MPyV (MOI = 1 pfu per cell), and the total DNA was extracted at 24 or 40 hpi. Infected cells (1 × 10^5^ to 3 × 10^5^) were pelleted by centrifugation (300 ***g***, 5 min, 20 °C), resuspended in 100 μL of lysis buffer [50 mm Tris–HCl (pH 8.0), 5 mm EDTA (pH 8.0), 1% Tween 20, proteinase K (80 μg mL^−1^)] and lysed for 1 h at 55 °C. The suspension was then incubated at 95 °C for 10 min to inactivate proteinase K, diluted 100× in H_2_O and 1 μL was used as a template for subsequent qPCR. Viral DNA was amplified by qPCR using the primer set for MPyV‐E (5′‐GCTGACAAAGAAAGGCTGCT‐3′ and 5′‐CCTTTGTCTGGGTGCAGTAG‐3′), and host cell DNA was amplified using the primer set for the single copy *p53* gene (5′‐GACTTGGGCTTTGGTGTTGG‐3′ and 5′‐ACCTTGATGATGGCTGTGGA‐3′). Quantification of the PCR products in real time was performed in a Light Cycler 480 II (Roche) using the Light Cycler 480 SYBR Green I Master Kit, according to the manufacturer's protocol. Quantification of target gene expression was performed using LightCycler 480 II software based on the relative quantification method, which determined the concentration of target MPyV genome amplicons normalised to the reference *p53* gene.

### Statistical analysis

Data are represented as the mean values from four independent experiments, unless otherwise stated, and the error bars represent the standard deviation. Student's *t*‐test was performed where indicated, using graphpad prism software, version 6.0 (GraphPad Software, La Jolla, CA, USA). *P < 0.05; **P < 0.01; ***P < 0.001 and ****P < 0.0001 were considered significant.

## Conflict of interest

The authors declare no conflict of interest.

## Author contributions

Planned experiments: KB and LH. Performed the experiments: KB, BR, SŽ, VŠ and LH. Analysed the data: KB, BR, SŽ, VŠ and LH. Wrote the paper: LH, KB and JF.

### Peer review

The peer review history for this article is available at https://www.webofscience.com/api/gateway/wos/peer-review/10.1111/febs.17275.

## Data Availability

The data that support the findings of this study are available from the corresponding author upon reasonable request.
